# Maintenance and Reversibility of Paroxysmal Atrial Fibrillation in JDP2 Overexpressing Mice

**DOI:** 10.3390/cells14141079

**Published:** 2025-07-15

**Authors:** Gerhild Euler, Jacqueline Heger, Marcel Rossol, Rainer Schulz, Mariana Parahuleva, Jens Kockskämper

**Affiliations:** 1 Institute of Physiology, Justus Liebig University, 35392 Giessen, Germany; jacqueline.heger@physiologie.med.uni-giessen.de (J.H.); rainer.schulz@physiologie.med.uni-giessen.de (R.S.); 2 Biochemical-Pharmacological Centre (BPC) Marburg, Institute of Pharmacology and Clinical Pharmacy, University of Marburg, 35043 Marburg, Germany; rossolm@staff.uni-marburg.de (M.R.); jens.kockskaemper@staff.uni-marburg.de (J.K.); 3 Internal Medicine/Cardiology and Angiology, University Hospital of Giessen and Marburg, 35033 Marburg, Germany; mariana.parahuleva@prof-parahuleva.de

**Keywords:** atrial hypertrophy, calcium, connexin, ECG, JDP2, paroxysmal atrial fibrillation, SERCA2a, ryanodine receptor

## Abstract

Heart-specific overexpression of transcriptional regulator JDP2 (jun dimerization protein 2) for 5 weeks provokes paroxysmal atrial fibrillation (AF) in mice. We now investigated whether AF and atrial remodeling will be reversible upon termination of JDP2 overexpression, and whether paroxysmal AF converts to permanent AF in the presence of maintained JDP2 overexpression. Cardiac-specific JDP2 overexpression for 5 weeks, resulting in paroxysmal AF, was either continued or repressed via a tet-off system for another 5 weeks. ECGs were recorded weekly. Thereafter, heart and lung weights, and atrial mRNA and protein expression were determined. Extending JDP2 overexpression did not aggravate the AF phenotype, still paroxysmal AF, prolongation of PQ intervals, and atrial hypertrophy were present. This phenotype was completely reversible upon cessation of JDP2 overexpression. A massive downregulation of connexin40 and calcium handling proteins, including SERCA2a, calsequestrin, and ryanodine receptor, was observed in atria after prolonged JDP2 overexpression. In conclusion, atrial remodeling and paroxysmal AF under JDP2 overexpression are not sufficient to maintain or aggravate AF in the absence of JDP2. The comparison of the two groups indicates that the downregulation of calcium proteins and connexins is an important factor in the maintenance of the disease.

## 1. Introduction

AF is the most common sustained cardiac arrhythmia worldwide, with an estimated global prevalence of 50 million people in 2020 [1]. Patients with paroxysmal atrial fibrillation often develop persistent or permanent atrial fibrillation, which is associated with an increased risk of stroke [2]. It is assumed that cardiac remodeling processes in the atria contribute to the progressive deterioration of atrial function and that there is a point at which the progression of the disease can no longer be reversed. But also in patients with paroxysmal AF, treated with anti-arrhythmic drugs and catheter ablation, some patients experience recurrence of atrial fibrillation [3]. Therefore, identification of new interventions that slow or reverse AF is important.

To study the mechanisms that contribute to the development of the disease, animal models of AF can be applied. In addition to large animal models, studies in transgenic mice that develop spontaneous occurrence of AF are now available [4]. One of these models is cardiac-specific JDP2-overexpressing mice, initially described by Kehat et al. [5]. JDP2 acts as a transcription regulator on diverse levels, i.e., as an inhibitor of AP-1-mediated transcription or as a chromatin remodeler [6]. JDP2-overexpressing mice develop paroxysmal AF within 4–5 weeks in juvenile or adult mice [5,7]. Interestingly, ventricular dysfunction precedes AF in adult mice. A decrease in ejection fraction and impaired contractile function of isolated ventricular cardiomyocytes were observed after only 1 week of JDP2 overexpression, while paroxysmal AF occurred after 5 weeks of JDP2 overexpression [8,9]. Paroxysmal AF was accompanied by atrial hypertrophy, prolongation of PQ and QRS intervals in ECG recordings, fibrosis, reductions in expression of connexin 40 and calcium handling proteins. Thus, after 5 weeks of JDP2 overexpression, atrial remodeling was evident in adult mice. Interestingly, JDP2 is increased in patients after myocardial infarction [10] and was identified as a prognostic marker for heart failure development after myocardial infarction in humans [10].

Since in humans, the progressive nature of the arrhythmic substrate is a major problem of AF treatment [11], we speculated that, also in JDP2-overexpressing mice, a similar disease progression may be observed. We therefore investigated whether the processes of cardiac remodeling are sufficient to promote further progression of atrial dysfunction and whether paroxysmal AF develops into persistent or permanent AF. For this analysis, JDP2 was overexpressed for the full 10 weeks (5 + 5 weeks) to determine if the continuous presence of JDP2 aggravates the cardiac phenotype. Another aim of the study was to clarify whether or not AF and atrial remodeling will be reversible upon termination of JDP2 overexpression. Since JDP2 overexpression was under the control of a tetracycline-regulated α-MHC promoter, cardiac-specific expression of JDP2 could be repressed by doxycycline feeding. After 5 weeks of JDP2 overexpression in adult mice leading to paroxysmal AF [7], overexpression of JDP2 was interrupted for another 5 weeks, and ECG recordings and expression analyses were performed.

## 2. Materials and Methods

### 2.1. Heart-Specific JDP2-Overexpressing Mice

The investigation conforms to Directive 2010/63/EU of the European Parliament. Animal studies were approved by the Regierungspräsidium Gießen (07.02.2022, registration-no V 54—19 c 20 15 h 01 MR 20/1 Nr. G 69/2021).

JDP2-mice, which are crossbreedings of *C57BL/6 J* and *FVB/N mice*, were generated in the group of A. Aronheim (Haifa, Israel). They are double-transgenic and carry the *JDP2* gene (*tetO*) and a transactivator gene (*tTA*) under control of the cardiac-specific *α-MHC* promoter. The genotype *tTA/tetO* identifies JDP2-overexpressing mice. Littermates that did not overexpress JDP2 with the genotypes *0/0*, *tTa/0*, and *tetO/0* were used as controls (wild type, WT).

**Figure 1:**
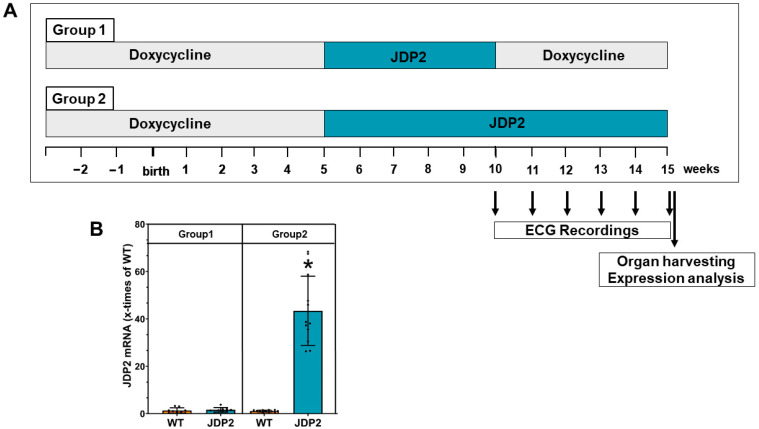
Experimental design of JDP2 overexpression. (**A**) Mice were fed with doxycycline diet until 5 weeks of age to suppress JDP2 overexpression in double transgenic mice. Then JDP2 overexpression was started by switching to standard diet. In group 1, JDP2 overexpression was stopped after 5 weeks, while in group 2, JDP2 overexpression was maintained over 10 weeks. WT mice received the same feeding protocols. ECG recordings were performed every week, starting at the time point of 5-week JDP2 overexpression. At the end of the experiments, hearts were removed to perform weight and expression analyses. (**B**) JDP2 mRNA expression was determined by real time RT-PCR (* *p* < 0.05 vs. WT, n = 9–13).

For suppression of JDP2 during embryonic and juvenile development of mice, breeding pairs and newborn mice were fed with doxycycline until five weeks after birth, followed by heart-specific JDP2 overexpression for up to 5 weeks in the absence of doxycycline feeding. After this time, one group was switched to a doxycycline diet for a further 5 weeks, which led to the cessation of JDP2 overexpression (group 1). The second group (group 2) was held for another 5 weeks without doxycycline feeding, resulting in continuous JDP2 overexpression over 10 weeks. WT mice received a doxycycline-diet for the same time as JDP2-overexpressing mice in each group (Figure 1A).

### 2.2. Electrocardiography Recordings

The ecgTUNNEL system (emkaTechnologies, Paris, France) was used to record ECGs in conscious mice. Weekly recordings were performed 5 weeks after the onset of JDP2 overexpression for 5 weeks. ECG analysis was carried out with the ecgAUTO software (v3.3.5.12, emkaTechnologies, Paris, France) using the shape recognition technique. According to Ni and colleagues [12], spontaneous AF was defined as the absence of P waves associated with an irregular heart rate lasting for more than 10 s. Duration of atrial fibrillation was determined as the sum of atrial fibrillation episodes within the 30-min recording time, and the incidence of atrial fibrillation was calculated as the percentage of mice in which atrial fibrillation was detected.

### 2.3. Isolation of Organs and Weight Determination

Euthanasia was performed under isoflurane inhalation (5% isoflurane) by cervical dislocation. Hearts and lungs were excised immediately. After wash-out of blood with ice-cold 0.9% NaCl solution, organ weights were determined. Tissues were frozen in liquid nitrogen and stored at −80 °C.

### 2.4. Real Time RT-PCR

Trizol (Invitrogen, Waltham, MA, USA) was used for the isolation of total RNA from atrial tissue. After DNAse treatment, RNA was reverse transcribed with QuantiTect Reverse Transcription Kit from Qiagen (Hilden, Germany). Real time RT-PCR was performed using SYBR Green fluorescence of the Biorad detection system (Bio-Rad, Hercules, CA, USA). The linear amplification range was tested for each primer pair. RNA expression was determined according to the 2−ΔΔCt method as described [13]. *GAPDH* was used as a housekeeping gene. Primer sequences are listed in Table 1.

### 2.5. Western Blots

Immunoblotting of atrial tissue lysates was performed similarly to procedures described previously [7,14]. Gradient gels (4–20% Mini-PROTEAN^®^ TGX^TM^ Precast Protein Gels, #4561093, BioRad, Hercules, CA, USA) or Tris Tricine gels (SERCA2a, PLB) were used for the separation of proteins. Gels were loaded with equal amounts (20 μg) of protein. In case of ryanodine receptor 2 (RyR2) detection, 30 μg of protein was used. Membranes were cut between protein bands of interest, so that we could detect various proteins on one membrane (and hence save sample material). The following primary antibodies were used (catalogue number, company, dilution): anti-Cav1.2 (CACNA1C) (#ACC-003, Alomone Labs, Jerusalem, Israel, 1:1000), anti-Ryanodine Receptor (C3-33) (#MA3-916, Thermo Fisher, Waltham, MA, USA, 1:1250), anti-Ryanodine Receptor 2 (pSer2808) pAb (#A010-30, Badrilla, Burlington, NC, USA, 1:1000), anti-Ryanodine Receptor 2 (pSer2814) pAb (#A010-31AP, Badrilla, Burlington, NC, USA, 1:500), anti-SERCA2 ATPase antibody (SERCA2a pAB SERUM) (#A010-20, Badrilla, Burlington, NC, USA, 1:5000), anti-Phospholamban (PLN, PLB) mAb (clone A1) (#A010-14, Badrilla, Burlington, NC, USA, 1:1000), anti-Calsequestrin polyclonal antibody (#PA1-913, Thermo-Fisher, Waltham, MA, USA, 1:2000), anti-connexin40 polyclonal antibody (#36-4900, Invitrogen, Waltham, MA, USA, 1:1000), anti-GAPDH (mAb (6C5), Calbiochem, Darmstadt, Germany, 1:50,000), anti-Vinculin antibody, mouse monoclonal, (#V9264, Sigma-Aldrich, St. Gallen, Swiss, 1:20,000). GAPDH or vinculin was used for normalization of expression.

### 2.6. Statistical Analysis

Mean ± SD was calculated for data presentation. Western blot quantifications are shown as mean ± SEM. Normally distributed data were analyzed with an unpaired 2-tailed Student’s *t*-test or ANOVA. Non-normally distributed data on AF duration were analyzed with the Mann-Whitney U test. For group comparison, Fisher’s exact test was used as indicated. GraphPad Prism (version 8 and 10, La Jolla, CA, USA) or SPSS (version 29; SAS Institute Inc., Cary, NC, USA) were used for those calculations. A *p* value < 0.05 was considered statistically significant.

## 3. Results

### 3.1. Prolonged Conduction Times and Atrial Fibrillation Under Prolonged JDP2 Overexpression

**Figure 2:**
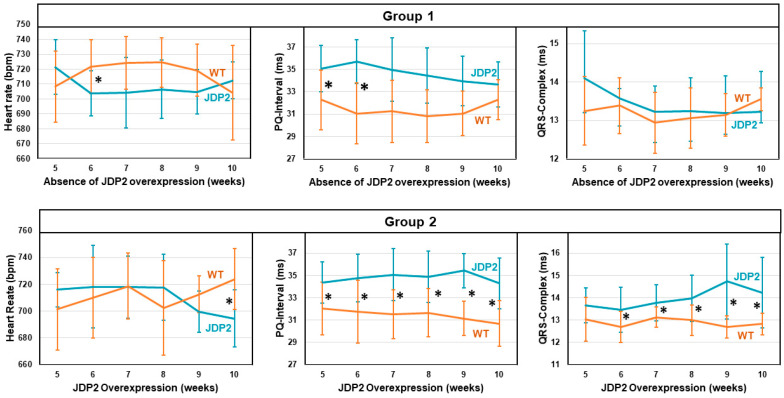
Conduction defects under prolonged JDP2 overexpression. ECGs were recorded over 30 min after 5 weeks of JDP2 overexpression, and then every week up to week 10, either in absence (group 1) or presence of JDP2 overexpression (group 2) (* *p* < 0.05 vs. WT, n = 9–12).

**Figure 3:**
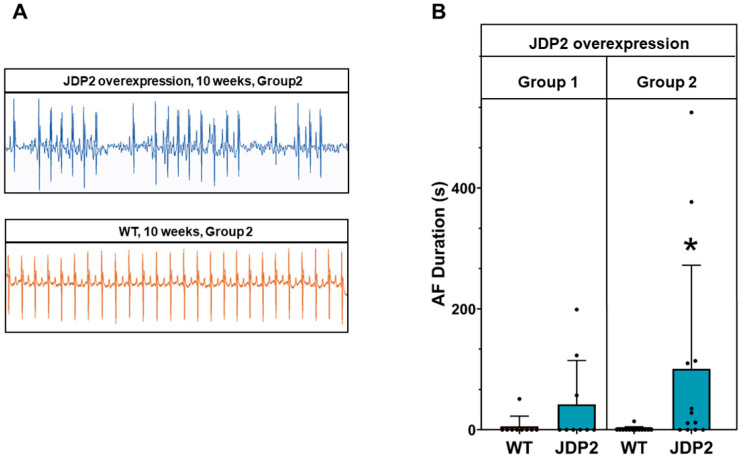
Paroxysmal atrial fibrillation under prolonged JDP2 overexpression. ECGs were recorded over 30 min after 5 weeks of JDP2 overexpression, and then every week up to week 10, either in absence (group 1) or presence of JDP2 overexpression (group 2). (**A**) Paroxysmal atrial fibrillation in mice overexpressing JDP2 for 10 weeks (**upper panel**), compared to sinus rhythm in WT mice (**lower panel**). (**B**) Mean duration times of atrial fibrillation (* *p* < 0.05 vs. WT, n = 9–13).

JDP2 mice and WT littermates were fed with a doxycycline diet for 5 weeks, followed by standard chow for another 5 weeks to induce JDP2 overexpression in double transgenic mice (Figure 1A). At this time point (5 weeks after the start of JDP2 overexpression), the first ECG measurements were performed. Similar to our previous findings [6], JDP2 overexpression provoked prolongation of the PQ interval (Figure 2) and paroxysmal AF. To test whether these effects are reversed upon cessation of JDP2 overexpression or whether they are maintained or aggravated with prolonged JDP2 overexpression, we divided the animals into two groups. One group of animals received doxycycline again to stop JDP2 overexpression for another 5 weeks (group 1). In this group, mRNA expression of JDP2 was normalized within these 5 weeks (1.4 ± 0.5 times compared to WTs, n.s., n = 9) (Figure 1B). The other group received standard chow to maintain JDP2 overexpression for a further 5 weeks (group 2), which resulted in 41 ± 0.7-times increased JDP2 mRNA expression (*p* < 0.05, n = 12–13) (Figure 1B). ECG recordings were performed every week in both groups. Interestingly, without further JDP2 overexpression (group 1), all changes in ECG recordings declined over time. At the end of the measurements, after 5 weeks without JDP2 overexpression, there were no obvious differences in ECG recordings compared to WT animals (Figure 2), and the average duration of AF within the 30 min recording time declined to 42 ± 68 s (n.s. versus WT, Fisher’s exact test) (Figure 3B). In the second group, with prolonged JDP2 overexpression, PQ time prolongation was apparent throughout the measurements, and widening of the QRS complexes occurred after 6 weeks of JDP2 overexpression and remained present in the following weeks (Figure 2). Paroxysmal AF with an average AF duration of 101 ± 163 s compared to 1 ± 3 s in WT was apparent (Figure 3A,B). Comparing JDP2 overexpressing mice of groups 1 and 2 revealed a decline in AF duration in group 1 (*p* < 0.05, n = 9–12). The incidence of AF (67%, i.e., 8 out of 12 animals developed AF) was significantly increased upon prolonged JDP2 overexpression (group 2) compared to WTs (8%, i.e., 1 out of 13 animals developed AF) (*p* < 0.05, Fisher’s exact test).

### 3.2. Hypertrophy of Atria and Ventricles Under Prolonged JDP2 Overexpression

**Figure 4:**
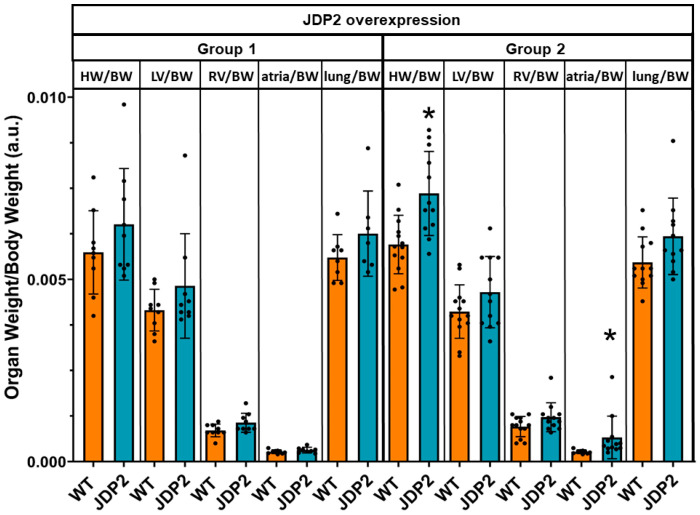
Organ weights in JDP2 mice. Lungs and hearts were harvested from mice after 5 weeks of JDP2 overexpression, followed by 5 weeks without overexpression (group 1) or another 5 weeks with JDP2 overexpression (group 2). The total heart weight (HW) and weights of left and right ventricles (LV and RV), atria, and lungs were determined and related to the body weight (BW) (* *p* < 0.05 vs. WT, n = 9–13).

**Figure 5:**
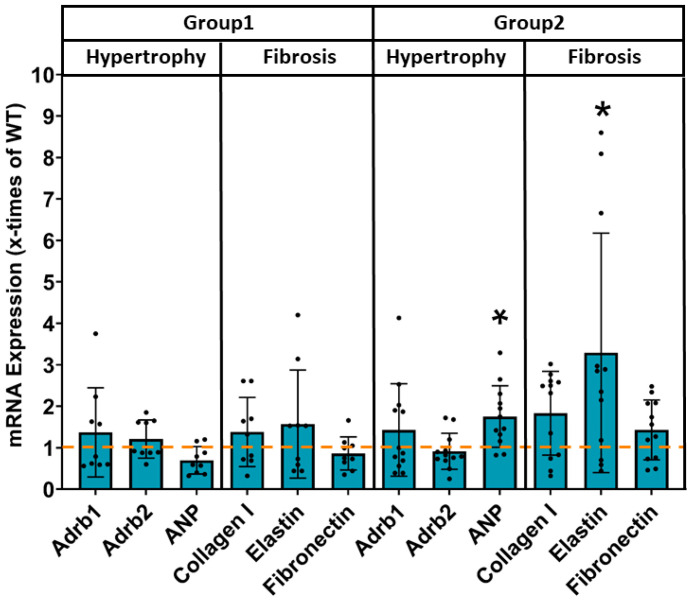
Induction of hypertrophic and fibrotic mRNA expression under prolonged JDP2 overexpression. mRNA expression in atria of mice after 5 weeks of JDP2 overexpression, followed by 5 weeks without overexpression (group 1) or another 5 weeks with JDP2 overexpression (group 2) was determined by real time RT-PCR (* = *p* < 0.05 vs. WT, n = 9–13). The dashed line indicates the mRNA expression level in WTs.

At the end of the experiments, hearts and lungs were removed in order to determine organ weights as an indicator of detrimental hypertrophic enlargement or lung congestion as a sign of heart failure, as well as expression of hypertrophic and fibrotic marker genes was analysed. As depicted in Figure 4, organ weights in group 1 with discontinued JDP2 overexpression were not significantly increased, and no changes in marker gene expressions were detected compared to WT (Figure 5). However, in group 2 with prolonged JDP2 overexpression heart weight/body weight ratio increased to 0.0074 ± 0.0011 compared to 0.0060 ± 0.0008 in WT (n = 12–13, *p* < 0.05), and atria weight/body weight ratio increased to 0.00066 ± 0.0006 compared to 0.00027 ± 0.00005 in WT (n = 12–13, *p* < 0.05). In addition, an increased mRNA expression of the hypertrophic marker gene ANP and pro-fibrotic elastin was evident (Figure 5).

### 3.3. Dysregulated mRNA Expression of Calcium Handling Proteins and Connexins Under Prolonged JDP2 Overexpression, but No Change of Inflammatory Parameters

**Figure 6:**
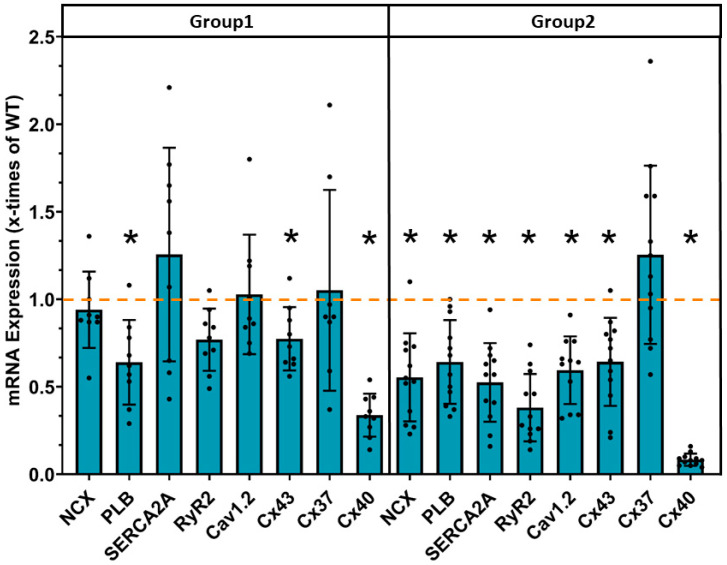
Decreased mRNA expression of various calcium-handling proteins and connexins under prolonged JDP2 overexpression. mRNA expression in atria of mice after 5 weeks of JDP2 overexpression, followed by 5 weeks without overexpression (group 1) or another 5 weeks with JDP2 overexpression (group 2) by real time RT-PCR (* = *p* < 0.05 vs. WT, n = 9–13). The dashed line indicates the mRNA expression level in WTs.

Disturbances in calcium handling and intercellular communication via gap junctions are the main contributors to cardiac arrhythmias. In adult mice overexpressing JDP2 for 5 weeks, we detected downregulation of calcium handling proteins and connexion 40 previously [8]. Interestingly, the decrease in mRNA expression of calcium-handling proteins was largely reversed in the absence of JDP2 overexpression (Figure 6). After 5 weeks in the absence of JDP2 overexpression (group 1), only a reduced mRNA level of PLB was present. Conversely, 5 weeks after cessation of JDP2 overexpression, not only connexin 40 but also connexin 43 mRNA was downregulated (Figure 6). In contrast to group 1, persistent overexpression of JDP2 in group 2 led to a strong decrease in mRNA expression of the calcium-handling proteins NCX, PLB, SERCA2A, RyR2, and Cav1.2 (Figure 6). Furthermore, the mRNA of connexin 43 was downregulated to a similar extent as in group 1 (Figure 6), and for connexin 40, the decrease in the mRNA level was more severe than in group 1 (n = 9–13, *p* < 0.05 vs. group 1).

**Figure 7:**
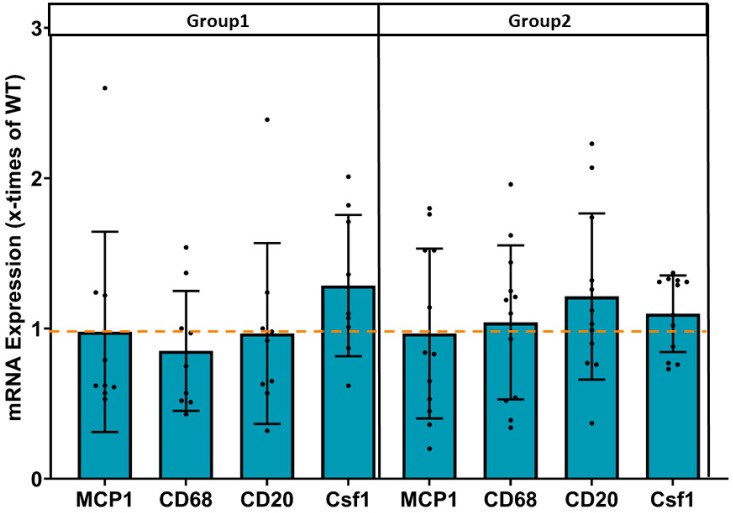
No change in inflammatory marker gene expression. mRNA expression of the inflammatory marker genes in atria of mice after 5 weeks of JDP2 overexpression, followed by 5 weeks without overexpression (group 1) or another 5 weeks with JDP2 overexpression (group 2) by real time RT-PCR (n = 9–13). The dashed line indicates the mRNA expression level in WTs.

In our previous studies in adult mice overexpressing JDP2 for 5 weeks [7], enhanced mRNA expression of several pro-inflammatory genes was detected. However, the analysis of these marker genes in groups 1 and 2 revealed a consistent decline in mRNA expression to WT levels of all pro-inflammatory marker genes (Figure 7). This decline was independent of the presence or absence of JDP2 overexpression. The transient, non-persistent increase in pro-inflammatory marker genes that occurred only at the beginning of JDP2 overexpression [7] indicates that inflammation is an early event in this model of AF.

### 3.4. Progressive Alterations in the Expression and Phosphorylation of SR Calcium Handling Proteins Under Prolonged JDP2 Overexpression Are Completely Reversible upon Withdrawal of JDP2

**Figure 8:**
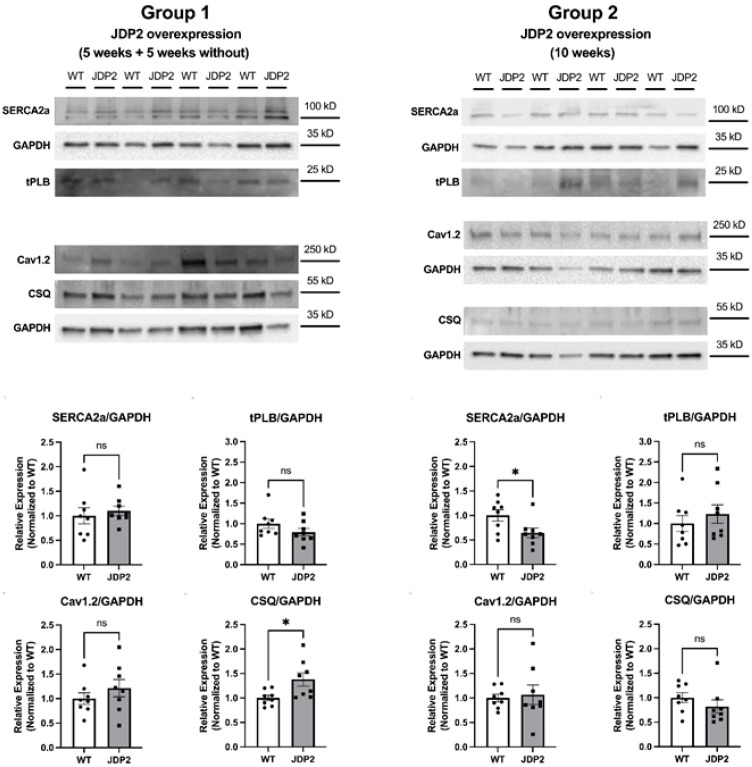
Expression of various calcium handling proteins with and without prolonged JDP2 overexpression. Original western blots and quantified data from atria isolated from mice after 5 weeks of JDP2 overexpression, followed by 5 weeks without overexpression (group 1) or another 5 weeks with JDP2 overexpression (group 2) (n = 8 per group; * *p* < 0.05, ns = non-significant). GAPDH was used as a loading control for normalization.

**Figure 9:**
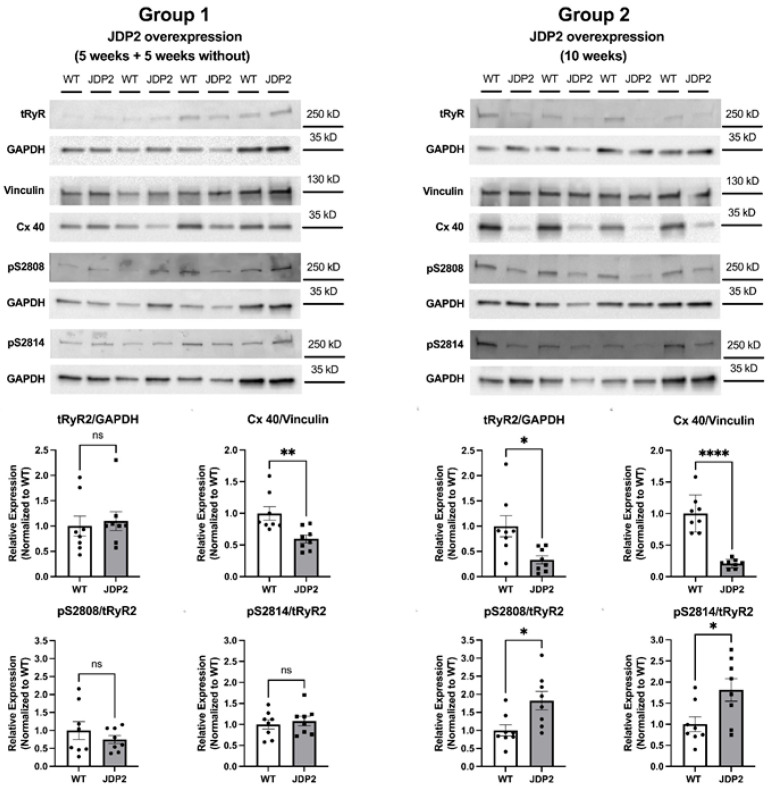
RyR expression and phosphorylation, and connexin 40 expression with and without prolonged JDP2 overexpression. Original western blots and quantified data from atria isolated from mice after 5 weeks of JDP2 overexpression followed by 5 weeks without overexpression (group 1) or another 5 weeks with JDP2 overexpression (group 2) (n = 8 per group; * *p* < 0.05; ** *p* < 0.01; **** *p* < 0.0001, ns = non-significant). GAPDH or vinculin was used as a loading control for normalization. Note that the GAPDH loading control shown for pS2808 is identical to the one shown in Figure 8 for CSQ, as the proteins were derived from the same membrane.

Altered expression and phosphorylation of calcium-handling proteins have been observed in atrial myocardium from animal models of AF as well as in human AF. Previously, we found reduced expression of SERCA2a and reduced phosphorylation of RyR2 at S2808 after 5 weeks of JDP2 overexpression [6]. Here, we studied the expression of Cav1.2, the sarcolemmal L-type calcium channel, and of SR calcium handling proteins SERCA2a, calsequestrin (CSQ), phospholamban (PLB), and RyR2 (Figure 8 and Figure 9). During prolonged overexpression of JDP2 for 10 weeks, expression of Cav1.2, CSQ, and PLB remained unaltered (Figure 8). Expression of SERCA2a was reduced by ≈35% (Figure 8), similar to what was observed after 5 weeks of JDP2 overexpression [6]. Furthermore, expression of RyR2 was drastically reduced by ≈67% in the JDP2 group (Figure 9). In addition, phosphorylation of RyR2 at S2808 and S2814 was also less pronounced in JDP2 samples when compared with WT samples (Figure 9). However, normalization to the expression of RyR2 (which was greatly reduced) revealed significantly larger (plus ≈ 80%) phosphorylation of RyR2 at serines 2808 and 2814 in the JDP2 group (Figure 9). Astonishingly, after 5 weeks of absence of JDP2 overexpression (group 1), all changes in expression and phosphorylation of SR calcium handling proteins were completely reversible (Figure 8 and Figure 9). Thus, no differences were observed in the expression of Cav1.2, SERCA2a, PLB, and RyR2 and in the phosphorylation of RyR2 at S2808 and S2814 between the JDP2 and the WT group under these conditions. Interestingly, CSQ exhibited slightly elevated expression now (plus ≈ 38%) in the JDP2 withdrawal group (Figure 8), but this was the only detectable difference in calcium handling proteins.

A key finding from previous studies on juvenile JDP2-overexpressing mice was a large down-regulation of Cx40 in the atria, which was partly reversible upon withdrawal of JDP2 [4]. In our mice, JDP2 overexpression for 10 weeks caused down-regulation of Cx40 by ≈80% (Figure 9). On the other hand, mice with 5 weeks of JDP2 overexpression followed by 5 weeks of JDP2 withdrawal exhibited only ≈40% down-regulation of Cx40 (Figure 9), suggesting that the JDP2 effect on Cx40 expression is, at least in part, reversible.

## 4. Discussion

The main findings of this study are that paroxysmal AF, which develops under short-term (5 weeks) heart-specific JDP2 overexpression, does not turn into persistent AF under prolonged (10 weeks) JDP2 overexpression. Interestingly, abrogation of JDP2 overexpression reverses the phenotype. AF declines, and other detrimental cardiac parameters, like cardiac hypertrophy or prolongation of PQ- or QRS-times, are no longer detectable following the absence of JDP2 overexpression. This indicates that the structural/molecular changes that occur when JDP2 is overexpressed in the heart are reversible and not sufficient to maintain or exacerbate the cardiac phenotype.

Interestingly, in humans, increased JDP2 levels are associated with progression to heart failure after myocardial infarction [10]. Also, in other studies, elevated JDP2 levels are found after myocardial infarction [9]. After myocardial infarction, not only ventricular but also left atrial remodeling develops and is an independent risk factor for mortality [15,16]. Therefore, the increase in JDP2 levels after myocardial infarction may contribute to the deterioration of cardiac function by promoting atrial remodeling and the development of atrial fibrillation. Furthermore, JDP2 is a transcription inhibitor of the CREB family, and an increased AF susceptibility was associated with decreased expression of the targets of the CREB family in humans [17]. Thus, JDP2 overexpression in mice may resemble phenotypes of human AF.

In our study, non-invasive ECG recordings were used for the detection of AF occurrence in conscious mice. This has the advantage that animals don’t have to undergo surgery for implantation of monitoring devices, nor that narcotics have any influence on heart rhythm. ECG recordings were performed every week for 30 min, and episodes with more than 10 s of absence of P waves and irregular heart rate were categorised as atrial fibrillation. This method resembles the most commonly performed intermittent rhythm monitoring techniques (short-duration ambulatory Holter ECG monitors) in patients. According to ESC guidelines, the time period of AF required for diagnosis on monitoring devices is not clear-cut. A standard 12-lead ECG measures 10 s [18], thus the same time as defined in our study. However, these monitoring strategies had poor sensitivity for detecting arrhythmia recurrence in AF ablation trials, and implantable cardiac monitors remain the gold standard for the detection of AF [3]. Therefore, due to intermittent measurements in our study, we may have missed some periods of AF, because not every case of paroxysmal AF will occur within the 30 min ECG measurement, and a type 2 error with detection of false-negative animals can occur. Despite this limitation, a significant decrease in AF incidence and duration of AF periods was demonstrated after abrogation of JDP2 overexpression. This indicates reversibility of AF under these conditions. Therefore, comparison of differential gene expression between the two experimental groups (group 1 with reversed AF under abrogation of JDP2 overexpression vs. group 2 with prolonged JDP2 overexpression) enables identification of factors associated with maintenance of AF. As outlined in the following paragraphs, inflammatory markers are found associated with the induction of AF, whereas connexins and calcium handling proteins are related to the maintenance of AF. Further investigations in this animal model are necessary to clarify functional links to AF in order to find new treatment options.

As recently shown by our group, paroxysmal AF in mice overexpressing JDP2 for 5 weeks was accompanied by prolongation of PQ-interval, cardiac hypertrophy, increased collagen expression, reductions in connexin 40 expression and calcium handling proteins, and the induction of pro-inflammatory markers [7]. Correlation of these parameters with the incidence of paroxysmal AF suggested that at least some of these parameters may cause AF. Furthermore, platelet activation driven by platelet-leukocyte/monocyte interaction contributes to AF-related thrombosis [7]. Interestingly, in mice with prolonged JDP2 overexpression and paroxysmal AF, still cardiac hypertrophy and a decline in mRNA expression of connexins and calcium handling proteins were pertinent. However, signs of inflammation were no longer detectable, since markers of monocytes/macrophages (CD68) and B-lymphocytes (CD20), as well as pro-inflammatory MCP-1, were no longer upregulated. This indicates that pro-inflammatory signals are not required to maintain AF. However, it cannot be ruled out that inflammation is an initial trigger for AF induction. This is discussed similarly in an editorial from Hiram [19], who describes the activation of damage-associated molecular patterns (DAMPs) and the resulting inflammation as a modulator of electrical conduction and generator of arrhythmic substrates. Interestingly, Murphy et al. [20] have shown that systemic inflammation in the lymphocyte adapter protein (LNK) knock-out mice increases susceptibility to AF. Scavenging of soluble tumor necrosis factor α (TNFα), but not interleukin 1β (IL1β) prevented AF susceptibility in these mice. In other models, activation of the NLR family pyrin domain containing 3 (NLRP3) inflammasome resulted in AF susceptibility [21]. In contrast to our mouse model, however, these mice did not develop spontaneous AF and required electrical stimulation in addition to the inflammatory trigger.

Furthermore, inflammation may trigger thrombogenic events [22]. Atrial cardiomyopathy, which precedes atrial fibrillation, also favours thrombogenic events in the atrial cavity. Conversely, pleiotropic effects of activated coagulation factors promote atrial remodelling [23]. In the ARISTOTLE trial, which investigated whether apixaban was non-inferior to warfarin in reducing the risk of stroke, any episode of atrial fibrillation (paroxysmal or permanent) lasting longer than one minute was used as an inclusion criterion for patients [24]. According to ESC guidelines, patients with paroxysmal or permanent AF are treated the same and receive chronic oral anticoagulation based on the presence of concomitant stroke risk factors [18]. Whether thrombogenicity or thrombus formation is enhanced in JDP2 overexpressing mice was not investigated in this study. Since in our animal model, none of the animals exhibited any obvious phenotype during daily scoring, and the mortality rate did not increase under JDP2 overexpression, we assume that there is no severe stroke-prone thrombus formation. However, the thrombogenic status of these mice should be analysed in future studies. Since thrombogenicity can increase at early time points of AF [25], it would be interesting to know whether changes can be observed in both test groups (reverse vs. paroxysmal AF).

Another interesting point is the finding that mRNAs of the gap junction proteins connexin 43 and 40 are downregulated in atria of mice with prolonged JDP2 overexpression, but also in the atria of mice with abrogated JDP2 overexpression. At the protein level, we observed a large downregulation of connexin 40 by ≈80% during prolonged JDP2 overexpression that was partly reversible (≈40% down-regulation) upon withdrawal of JDP2. Since in the latter situation, mice do not present AF, it can be concluded that a moderate downregulation of connexin 40 can be tolerated without AF development. However, under prolonged JDP2 overexpression, the increased PQ interval and AF are accompanied by a severe downregulation of connexin 40. Under these conditions, connexin 40 can no longer convey the easy and fast conduction via gap junctions in the atrial tissue and may thereby contribute to PQ prolongation and AF. Besides regulation of connexin expression, the lateralization of connexins, resulting in reduced connexin density at the intercalated disc [26], as well as their phosphorylation status, can modulate the channel function [27].

So, if inflammation is not involved in the maintenance of atrial fibrillation under JDP2 overexpression and the reduction of connexin 40 only partially contributes to the atrial fibrillation phenotype, what could be the main causes of AF in JDP2 mice? Comparing gene expression between mice with abrogated vs. prolonged JDP2 overexpression, the general decrease in mRNAs coding for calcium handling proteins, and the enhancement of marker genes for cardiac hypertrophy and fibrosis are striking, and highlight them as factors maintaining AF. All of them may also cause the slowing of the conduction times that are observed in ECGs from mice with prolonged JDP2 overexpression. In accordance with the increased hypertrophic and fibrotic markers, heart and atrial weight were increased only in mice with prolonged JDP2 overexpression.

Remodeling of calcium-handling proteins was also studied in more detail at the level of protein expression and phosphorylation. At variance with the mRNA data, in mice with prolonged JDP2 overexpression, some of the calcium handling proteins, i.e., Cav1.2 and PLB, exhibited unaltered protein expression despite significantly reduced mRNA expression. This finding underscores the fact that reduced mRNA levels do not necessarily translate into reduced protein expression and that cellular protein abundance may be regulated by means other than mRNA expression. This is particularly true for Cav1.2, where protein expression (and/or L-type calcium current) is often found to be much larger than would be expected from the reduced gene expression of the channel subunit α1C/*CACNA1C* [28,29,30]. SERCA2a and RyR2, on the other hand, showed reduced expression both at the mRNA and protein levels. Compared to the remodeling after 5 weeks of JDP2 overexpression [7], the reduction of SERCA2a protein levels remained rather constant (by ≈35–40%). RyR2, however, exhibited a dramatic down-regulation not observed after 5 weeks of JDP2 overexpression, indicating a clear progression of remodeling with increased duration of JDP2 overexpression. Moreover, phosphorylation levels of RyR2 changed with the duration of JDP2 overexpression. Whereas at 5 weeks of overexpression, RyR2 exhibited reduced phosphorylation at S2808 [7], after 10 weeks of JDP2 overexpression, RyR2 phosphorylation was greatly elevated both at S2808 and at S2814. The latter changes might be perceived as an attempt to increase RyR2 open probability in the face of the dramatic down-regulation of RyR2 protein levels. It is difficult to deduce the functional changes in SR calcium release from the above-mentioned alterations in calcium handling proteins. On the one hand, SR calcium load might be decreased because of the reduced expression of SERCA2a, and calcium transients might likewise be reduced because of decreased SR calcium load and reduced expression of RyR2. This would result in impaired atrial contraction. On the other hand, the greatly increased phosphorylation of RyR2 at both S2808 and S2814 augments RyR2 open probability and could increase spontaneous diastolic SR calcium release—even in the presence of decreased SR calcium load—thus increasing the risk of spontaneous atrial arrhythmias. Interestingly, reduced SERCA2a (by ≈40%) and unaltered PLB expression were also found in human paroxysmal AF, as well as unaltered L-type calcium current [30]. In contrast to the situation in JDP2-overexpressing mice, however, RyR2 expression was increased in human paroxysmal AF [31].

Atrial remodeling is an independent risk factor for AF in humans. Therefore, reversion of atrial remodeling should be an important aim in the treatment of AF patients. At the moment, comorbidities, rhythm and rate control, as well as avoidance of stroke and thromboembolism, are the main treatment targets in the ESC guidelines [18]. In some patients, this treatment also leads to a reduction in atrial remodeling. E.g., after pulmonary vein isolation by catheter ablation, which is an effective treatment option for rhythm control, reductions in atrial remodeling can be found in 63% of patients [32], or in HFpEF patients with AF, reverse remodeling of the left atrium was observed in 24.90% of patients after ablation [33]. The other part of the patients continued to show structural and functional atrial impairments and were therefore at a greater risk for AF recurrence. Structural remodeling is often associated with sustained rather than paroxysmal AF, representing a second association with failure of ablation. Furthermore, pulmonary vein isolation is not always possible or successful, as well as antiarrhythmic drugs are limited in their efficiency [34]. Therefore, additional therapeutic options are warranted. Since atrial remodeling is a complex process, involving dilatation, fibrosis, connexin dysregulation, or electrical disturbances, therapeutic options in this area could improve AF treatment. JDP2 mice now offer the opportunity to compare animals with ongoing paroxysmal and regressive AF. This may enable characterization of essential factors that are responsible for atrial remodeling and maintenance of AF.

Interestingly, all of the substantial alterations in calcium handling proteins observed in JDP2-overexpressing mice were completely reversible upon abrogation of JDP2 overexpression, as were the episodes of paroxysmal AF. This suggests that the calcium handling abnormalities were a major contributor to the maintenance of AF, presumably by triggering AF episodes through spontaneous SR calcium release events mediated by opening of highly phosphorylated RyR2. Functional data supporting this hypothesis, however, are lacking and should be the aim of future studies in JDP2-overexpressing mice.

Furthermore, the analyses of other parameters, like increased thrombogenicity, metabolic changes, or energetics, in future studies should complete the picture of contributors to JDP2-induced AF.

## 5. Conclusions

Paroxysmal AF, atrial remodeling, and changes in calcium handling proteins are reversible after elimination of the original trigger molecule JDP2 in mice. The comparison of animals with regressive AF and current AF makes it possible to narrow down factors that are important for AF maintenance, namely connexin downregulation, cardiac fibrosis, hypertrophy, and disturbed calcium handling. In further functional studies, this has to be proven before a therapeutic application to humans can be considered.

## Figures and Tables

**Table 1:** Primer sequences for real-time RT-PCR.

Primer	Sequences of Primer
Adrb1	Qiagen, QT00258692
Adrb2	5′-TGGTACCGTGCCACCCACAA-3′
	5′-AAGACCATCACCACCAGGGGCA-3′
ANP	5′-CTGCTAATCAGCCATGCAAA-3′
	5′-GATGGAGACCATCCTGGCTA-3′
Cav1.2	5′-CAGCCACTCTCCAGTCACTC-3′
	5′-CTGGAGTAGGGATGTGCTCG-3′
CD20	5′-CCTTTCCCAGCAGAGCCTAC-3′
	5′-TCATGATTTGGACAGCCCCC-3′
CD68	5′-ACTTCGGGCCATGTTTCTCT-3′
	5′-GCTGGTAGGTTGATTGTCGT-3′
Collagen1	5′-TTCTCCTGGRAAAGATGGTGC-3′
	5′-GGACCAGCATCACCTTTAACA-3′
Csf1r	5′-TCCACCGGGACGTAGCA-3′
	5′-CCAGTCCAAAGTCCCCAATCT-3′
Cx37	5′-ATAAAGGCACGAAGGGACCA-3′
	5′-GTCAAGTTGGCCCAGTTCTG-3′
Cx40	5′-AGGGCTGAGCTTGCTTCTTA-3′
	5′-TTAGTGCCAGTGTCGGGAAT-3′
Cx43	5′-GAAACAATTCCTCCTGCCGC-3′
	5′-AGTTGGAGATGGTGCTTCCG-3′
Elastin	5′-CTGCTGCTAAGGCTGCTAAG-3′
	5′-CCACCAACACCAGGAATGC-3′
Fibronectin	5′-ACAGAGCTCAACCTCCCTGA-3′
	5′-TGTGCTCTCCTGGTTCTCCT-3′
GAPDH	5′-TCCATGCCATCACTGCCACTC-3′
	5′-TGACCTTGCCCACAGCCTTG-3′
JDP2	5′-ATGATGCCTGGGCAGATCCCA-3′
	5′-TCACTTCTTGTCCAGCTGCTCC-3′
MCP1	5′-CCACAACCACCTCAAGCA-3′
	5′-TGAAAGGGAATACCATAACATC-3′
NCX	5′-CTACCAGGTCCTAAGTCAACAG-3′
	5′-TGCGTGCCTCTTCAAGATG-3′
PLB	5′-GCAATACCTCACTCGCTCGGCTATC-3′
	5′-TGGAGATTCTGACGTGCTTGCTGAG-3′
RyR	5′-AGCTGGAAGACCCTGCAATC-3′
	5′-ACCAGGCTGAAATATCCCCG-3′
SERCA	5′-TGACTGGTGATGGTGTGAATG-3′
	5′-GATGAGGTAGCGGATGAACTG-3′

## Data Availability

The original contributions presented in this study are included in the article. Further inquiries can be directed to the corresponding author.

## Abbreviations

The following abbreviations are used in this manuscript:

AF	Atrial fibrillation
CSQ	Calsequestrin
ECG	Electrocardiogram
JDP2	Jun dimerization protein 2
PLB	Phospholamban
RYR	Ryanodine receptor
WT	Wild type
